# Abcès de cornée: complication grave d'un traumatisme cornéen négligé et mal traité

**DOI:** 10.11604/pamj.2014.19.204.4392

**Published:** 2014-10-24

**Authors:** Amal Alouan, Rajae Daoudi

**Affiliations:** 1 Université Mohammed V Souissi, Service d'Ophtalmologie A de l'hôpital des spécialités, Centre Hospitalier Universitaire, Rabat, Maroc

**Keywords:** Abcès de cornée, traumatisme cornéen, œil, Corneal abscess, corneal trauma, eye

## Image en medicine

Un patient de 60 ans diabétique qui rapporte la notion de traumatisme cornéen de l’œil droit depuis 15 jours par une épine végétale lors des travaux de jardinage. Devant l'aggravation de la symptomatologie oculaire (larmoiement, photophobie) et l'apparition de douleur oculaire intense, il a pris un collyre corticoïde 3 jours avant sa consultation. L'examen ophtalmologique au niveau de cet œil trouve une acuité visuelle réduite à une perception lumineuse. La cornée est le siège d'une infiltration stromale (abcès) grossièrement ovalaire de 7mm*8mm, avec une large ulcération et un amincissement centrale. Le grattage cornéen a été effectué avant tout traitement et l’étude microbiologique a objectivé la présence d'amibes. L'infection cornéenne amibienne est l'apanage des porteurs de lentille de contact (90% des cas). Dans 10% des cas il s'observe après un traumatisme cornéen suivi à une exposition à de l'eau souillée ou des végétaux. L'atteinte cornéenne est tout d'abord épithéliale et si elle n'est pas prise en charge à temps, la kératite amibienne se développe progressivement pour atteindre le stroma cornéen. Le traitement antiamibien comporte une bithérapie locale et un traitement général pendant 4 mois. La corticothérapie est à proscrire au début en raison de l'immunodépression. 3 diagnostics différentiels: kératite bactérienne, kératite herpétique et kératite fongique.

**Figure 1:**
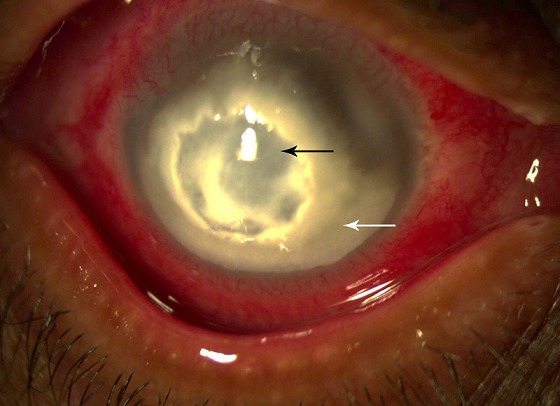
Kératite stromale (flèche blanche) et ulcère centrale (flèche noire)

